# Same same but different – A qualitative study on the development and maintenance of personal networks among German and international medical students

**DOI:** 10.3205/zma001204

**Published:** 2018-11-30

**Authors:** Timo Astfalk, Brigitte Müller-Hilke

**Affiliations:** 1 University Medical Center Rostock, Institute for Immunology, Research Group on Clinical Immunology, Rostock, Germany

**Keywords:** International medical students, cultural diversity, minorities, qualitative methods, grounded theory, social learning, social identity

## Abstract

**Introduction: **In addition to linguistic and cultural difficulties, problems with social integration are frequently found among international medical students. In contrast, the social interaction among German medical students is considered as non-problematic. Personal networks are considered as positively influencing factors for the learning environment of students as well as their academic performance and personal wellbeing. However, while general findings on the development and maintenance of personal networks among medical students are available, there is insufficient data on students with different nationality or cultural backgrounds. Therefore, we compared the perception of international and German medical students on the development and maintenance of personal networks and examined possible influencing factors.

**Methodology: **Following the principles of theoretical sampling in qualitative research, we recruited 17 international and 10 German preclinical medical students. The survey was conducted with semi-structured interviews, which focused on the participants’ experiences with the development and maintenance of personal networks in medical school. The coding and analysis of the interview transcripts was based on the principles of grounded theory.

**Results: **We show that German participants rated the network development in medical school much more positively than the international participants. This difference was influenced by: a different perception of affiliation to the group of medical students, a low academic benefit from contacts with international students, the socio-cultural framework of medical school as well as the individual strategies of international medical students in dealing with integration.

**Conclusion: **Our study extends existing insights into the development and maintenance of the social environment in medical school by understanding the perception of a subgroup of medical students. A better understanding of these social processes enables an effective evaluation of support and teaching for the subgroup of international medical students in Germany.

## Zusammenfassung

**Einleitung:** Soziale Integration stellt neben sprachlichen und kulturellen Schwierigkeiten ein häufiges Problemfeld für internationale Medizinstudierende dar. Das steht im Gegensatz zu der guten sozialen Integration unter deutschen Medizinstudierenden in ihren Jahrgängen. Dabei gilt eine gute Vernetzung im Studiengang als positiver Einflussfaktor auf das Lernumfeld, die Studienleistungen sowie das Wohlbefinden von Studierenden. Während aber qualitative Untersuchungen zur Bildung und Erhaltung der persönlichen Netzwerke im Medizinstudium im Allgemeinen vorliegen, fehlt die Betrachtung von sprachlich und kulturell heterogenen Untergruppen dieser Studierendenschaft. Wir verglichen daher die Wahrnehmung von internationalen und deutschen Medizinstudierenden zur Bildung und Erhaltung persönlicher Netzwerke im Studium und untersuchten mögliche Einflussfaktoren.

**Methodik: **Wir folgten einem qualitativen Forschungsdesign, bei dem 17 internationale und 10 deutsche vorklinische Medizinstudierende nach dem Prinzip des Theoretical Sampling rekrutiert wurden. Die Befragung erfolgte mittels halbstrukturierter Interviews, die auf die Teilnehmererfahrungen bzgl. Bildung und Erhaltung von persönlichen Netzwerken im Studium abzielten. Die Kodierung und Analyse der Interviewtranskripte richtete sich nach den Prinzipien der Grounded Theory Methodologie.

**Ergebnisse:** Deutsche Teilnehmende bewerteten die Netzwerkbildung im Studium im Allgemeinen deutlich positiver als die internationalen Teilnehmenden. Diese Bewertungsunterschiede wurden beeinflusst durch: eine unterschiedlich wahrgenommene Zugehörigkeit zur Gruppe der Medizinstudierenden, einen geringen akademischen Nutzen von Kontakten zu internationalen Studierenden, die soziokulturellen Rahmenbedingungen des Medizinstudiums sowie individuelle Strategien der internationalen Medizinstudierenden im Umgang mit der Integration in ihrem Jahrgang.

**Schlussfolgerung:** Unsere Studie erweitert bestehende Erkenntnisse zur Bildung und Erhaltung des sozialen Umfelds im Medizinstudium um die Einsicht in die Wahrnehmung einer Untergruppe von Medizinstudierenden. Ein besseres Verständnis dieser sozialen Prozesse ermöglicht eine effektive Evaluation von Unterstützungs- und Lehrangeboten für die Untergruppe der internationalen Medizinstudierenden in Deutschland.

## Introduction

International medical students worldwide [1], [2] as well as in Germany [3], [4], [5] show lower academic performance and a longer duration of study compared to the respective national groups. Medical schools [6], [7] and international students themselves [8] are aware of these academic difficulties as well as of the general problems in coping with the daily life and the integration into the student body. In the past, this was primarily explained through a language barrier among international medical students [9], [10]. However, this approach is increasingly being challenged, as it may not explain performance differences between different cultural groups with the same language [11], [12], [13]. Instead, the personal network and the learning environment, which students find in their medical schools has gained interest [14], [15], [16]. The personal surroundings may play an important role in coping with learning stress [17], [18], promoting wellbeing [19], [20] and also strengthening the development of a professional identity [21].

Creating such a supportive environment is generally considered uncomplicated for new medical students [22]. They commonly form a specialized disciplinary culture [23], which is highly valued by its members, but also perceived as exclusive by non-medical students [24]. Lovell [18] explains these phenomena through isolation processes as well as social support and control within the medical student body. They lead to the formation of a connected and shared social identity as medical students. This concept is based on the work of Tajfel [25] and Turner [26], who define social identity as an individuals’ knowledge of his group affiliation and its personal relevance [27].

Even though, international medical students share the same studies, they are considered to be less integrated within the medical student community. International students may have more difficulties in personal networking with fellow students and receive less support than German medical students [28]. Language barriers and cultural differences commonly create separate groups which can be seen in the social network of the medical student community [29], [30], [31].

Existing findings on group dynamics among medical students [32], [33] as well as the model for group formation in medical student communities proposed by Lovell [18] offer only insufficient explanation for these phenomena. At the same time, the theories of social identity used by Lovell [18] suggest effects through perceived group affiliations that need to be deepened [25], [26]. In this context, the present work pursues two central questions:

1. How do German and international medical students perceive the development and maintenance of their personal networks during their studies?
2. Which factors influence this perception?

## Methods

### Methodological approach

Due to the limited knowledge on the subject of research at German medical faculties, an exploratory approach was chosen [34]. The grounded theory methodology (GTM) of Corbin and Strauss allows such an inductive creation of hypotheses based on selected qualitative data [35]. This selection of data is not random and follows theoretical considerations on the research subject. In particular, the search for the widest possible variety of information, opinions and personal backgrounds of the study participants constitutes a central aim of data collection [34]. Although pre-planned study designs have been described with GTM [36], the puristic research process repetitively switches between data collection and analyses [35]. This makes it possible to recognize patterns by comparing contrasting cases. The saturation with such recurring elements in the data set marks the end of the research process [37]. In order to guarantee the quality of the results from the beginning, the research process is constantly reflected and documented through written memos [34], [38].

#### Data collection

The present study uses the data of 27 preclinical medical students, who were interviewed in the fall term of 2015 as part of a research project on social networks among medical students at the University Medical Center Rostock (UMR). The interviews were conducted with a semi-structured guide and later transcribed verbatim. The study participants were recruited via digital channels (facebook groups for medical students at UMR) as well as in person in the university library. Due to the research focus on international medical students, an initial question aimed for the nationality of the interested students. The study participants were chosen based on their nationality, first spoken language and socio-demographic background to enable the creation of a diverse sample. However, as international students from non-EU countries were underrepresented, they were contacted in an additional second step via personal contacts of the research team. All participants were in the preclinical part of their medical studies at UMR, which offers a standard medical course over 13 semesters [39]. Approximately 200 students begin their medical studies every year, with approximately one third of the students being from Mecklenburg-Vorpommern [40]. The age and gender distribution mostly correlate with the German average for medical students of 24 years and a proportion of 60% female students in Germany [41], [42]. Study participants include students within the designated period of study as well as resitters of the courses. We included a total of 27 students, 10 German participants, 9 from EU countries and 8 from non-EU countries. In most cases, the first spoken language and the country, in which the university entrance qualification was awarded, corresponded with the nationality. In one case an EU citizen had a German university entrance qualification. One participant had German citizenship, but named a first language from a non-EU country. Table 1 (Tab. 1) summarizes the demographic data of all study participants.

#### Data analysis

**Figure 1:**
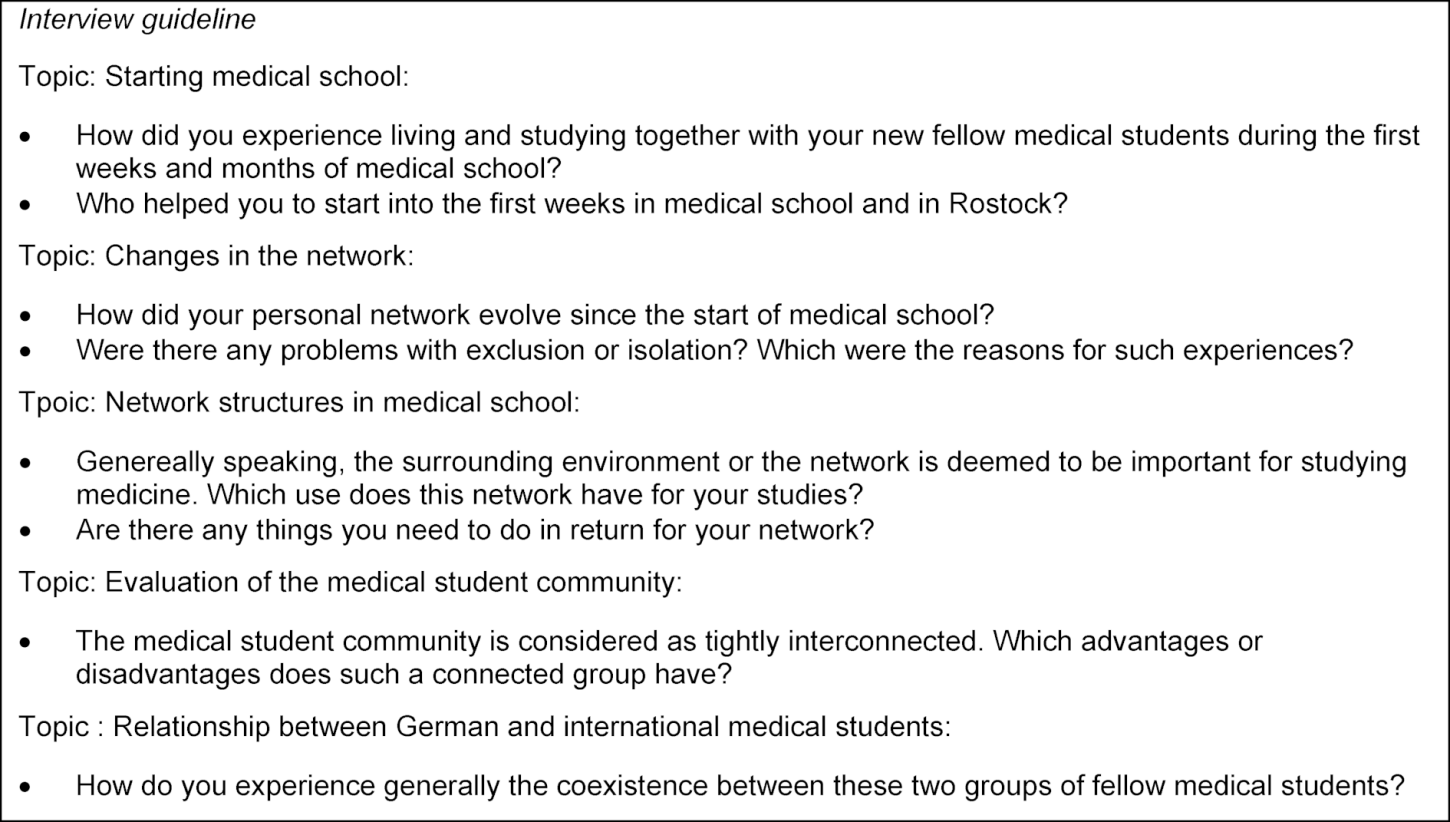
Interview guide. Over the course of the interview additional topics could be added in order to open up the guidline for new impressions of the study participants.

**Figure 2:**
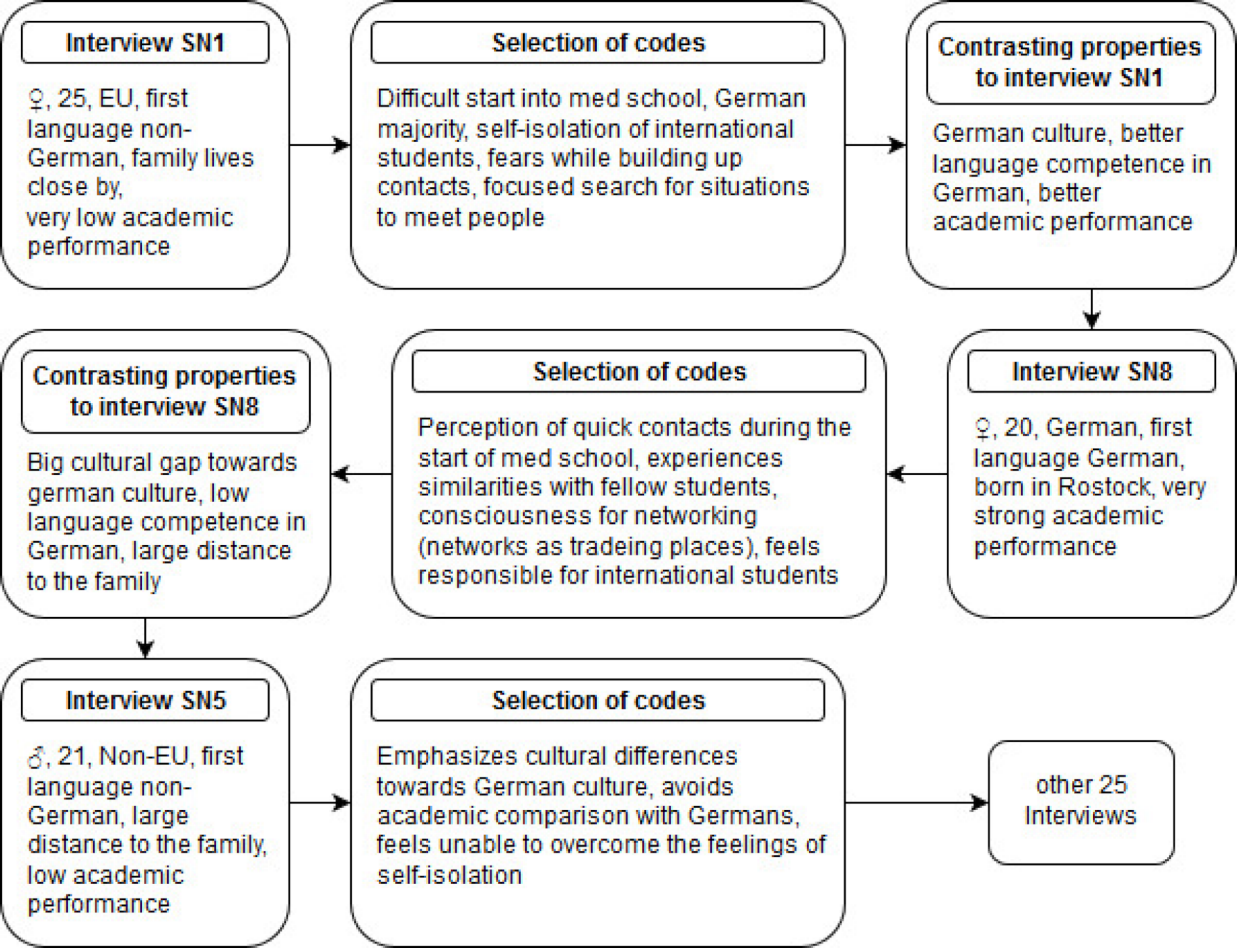
Excerpt from the process of data analysis through contrasting comparisons between interviews. Codes are derived from contrasting views and perceptions between different study participants (SN).

The interviews lasted an average of 23 minutes and were conducted by the first author, who was trained and experienced in grounded theory and in interview techniques. The interview guide focused on the development and maintenance of personal networks of study participants at the start of medical school. It offered the possibility for further inquiries beyond a fixed number of questions in order to enable reactions to statements of the students on new topics. The interview guide (see Figure 1 (Fig. 1)) is based on a literature selection on study problems of international medical students [10], [15], [43], [44], [45], [46], [47] and personal experiences of medical teachers at the UMR. The interviews took place in quiet places without influence of third parties. Afterwards they were transcribed verbatim and then transferred into the coding program MAXQDA 12 [48]. The order of the interview coding was chosen in such a way that certain characteristics of the study participants contradicted each other (e.g. first spoken language or academic performance). Through such comparisons we expected a strong contrast between study participants, who were then grouped based on similarities and opposites. Figure 2 (Fig. 2) illustrates the principle of this contrast coding. Contradictory cases as well as possible explanations were deliberately elaborated and discussed together with general coding memos by the research team. In addition, the results were discussed within the student research group on Medical Education at the Institute of Immunology.

#### Ethics

All participants were informed about the subject and aim of the study and gave written consent to participate. This study has been reviewed and approved by the UMR Ethics Committee (Proposal A 2015-0161).

## Results

Our results indicate distinct differences between German and international students in the challenges associated with development and maintenance of personal networks during the first semesters of medical school. German students describe the creation of relationships with fellow students mostly as a positive experience that was pleasant, quick and effortless. In contrast, international students report this process in a more negative way, with more difficulties in establishment of relationships and over all less contact to fellow students as well as feelings of isolation.

Yeah it was actually great. I was new to the place and didn't know anyone really. [...] It was definitely really nice at the beginning to be welcomed with open arms and to find so many friends. - German, M, SN11

It was actually really bad. I had absolutely no friends and, well what really bothered me was that everyone simply called me "the Spaniard", when they met me [changed nationality]. - EU, F, SN10

Out data analysis revealed a number of key topics that are relevant in regard to the influencing factors for these different views on network development and maintenance. They can be grouped into four general categories: different perception of group affiliation, unfavorable cost-benefit analysis, framework conditions of medical school and strategies for dealing with integration.

### Different perception of group affiliation

**Figure 3:**
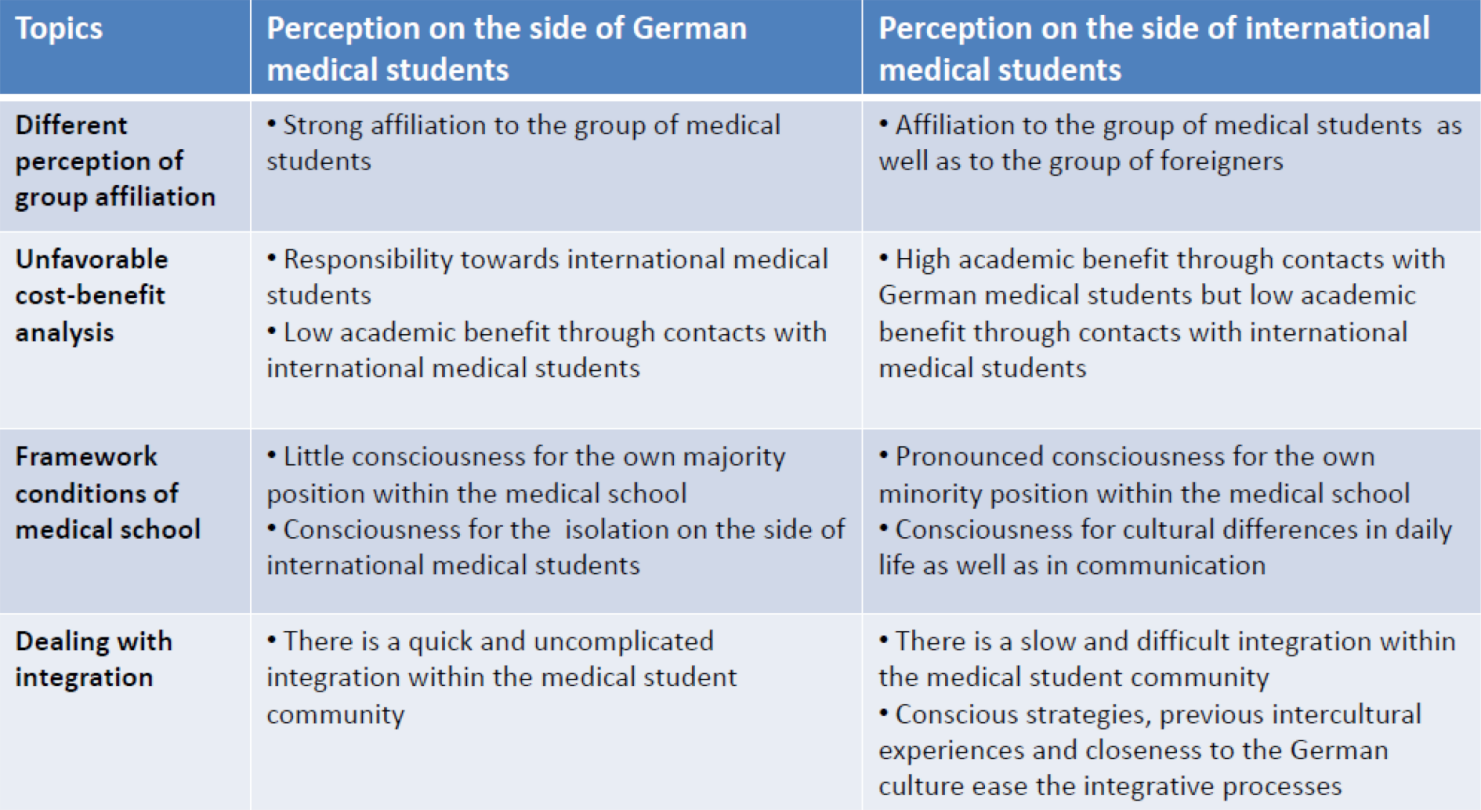
Differences in the perception between German and international medical students

All interviewed students emphasize group affiliation as a key element during the initial phase of medical school. While belonging to the group of medical students was omnipresent, international students additionally perceived their status as “foreign” as opposed to “domestic”. The role as medical students was generally perceived as positive. The separation from non-medical contacts was emphasized and attributed to the contents and framework conditions of the medical studies (see Figure 3 (Fig. 3)).

I believe that if you study medicine, then your life is only or almost only about medicine. And if we join into a small alliance as medical students it helps. [...] And we are so many. That is an important idea. - Non-EU, M, SN5

And you distinguish yourself from others a bit. Not as something better or worse, but you are your own circle of [medical] students. That's my impression. We noticed it immediately on the medical inauguration-weekend. To my knowledge, medicine is the first and only faculty with such a ritual. - German, M, SN27

However for international students the group affiliation as medical students has not the same degree of comprehensiveness. Their experience is additionally affected by the experience of being in the “foreigners”-group. This usually comes with responsibilities (e.g. appointments at the immigration office) and difficulties in everyday life (e.g. language barrier) and is thus often negatively connoted. This group affiliation was perceived by many as a new experience that sets them apart from the group of German students. Being German, however, did not result in an affiliation as “domestic” students.

At first I saw an extreme distinction that I am a foreigner and that language was a barrier. For the most of it. - EU, M, SN19

#### Unfavorable cost-benefit analysis

All students describe a change in the spirit within the medical student community after the first weeks of medical school. They describe a switch from initial cooperation between students towards a competitive orientation among each other. Over the course of the first weeks, the view on social contacts between students changes as well, as they imply not only benefits, but also social obligations (see Figure 3 (Fig. 3)).

Well I noticed it last year. This change from a party group towards a different one. Where you are focused on achievement. - German, M, SN27

We are a really competitive class, where everyone wants to rise up. - Non-EU, M, SN23

As a consequence, students evaluate which social contacts should be maintained during medical school. This process is often based on an academic benefit that can be drawn from the contacts. International students score relatively poorly on this cost-benefit analysis, as reported by both international and German students.

Yeah the problem is there are good things I can get from Germans but them? What can they get from me? For their studies? - Non-EU, M, SN9

I am really sorry, but it is also exhausting for oneself. I mean, when you also need to take care of someone else, because you do feel somewhat responsible for them. - German, F, SN8

The basis for the evaluation of social contacts is often the academic performance in medical exams. Often, the results of exams spread quickly within the medical student community despite privacy precautions. As a result, all students report a constant and unavoidable pressure to compare themselves and their academic performance.

I mean we all know almost all of our student IDs. So you definitely know what [grade] others have. - German, F, SN3

Not to say that we [the international students] perform worse, but they [German Students] are like: “she has more struggles than I and surely she is not as advanced on the subject as I am”. Therefore, they prefer talking with someone from Germany. - EU, F, SN20

#### Framework conditions of medical school

International students are a minority within the medical student community and our data indicates that they are well aware of their exposure within this asymmetric distribution. Although this exposure does not necessarily imply direct negative consequences, it does lead, from the point of view of some participants, to a sense of intimidation (see Figure 3 (Fig. 3)).

It is more difficult to build [a friendship] with Germans because they are the majority. They know each other just from seeing or from saying "hello" or such. [...] In comparison to us, it is the same, all foreigners know each other, but not all Germans. But the Germans also know all foreigners, because there are not so many. - Non-EU, M, SN23

It is noticeable as well that the international medical students are always among themselves. [...] I also think that international students are intimidated, because we might also be afraid - Non-EU, F, SN24

This intimidation among international students is also perceived by German students and often equated with less openness or even lack of interest for new contacts. 

I believe that most foreign students are very restraint [...] they are not open enough. [...] But, yeah I see it this way. That integration really fails on this point. - German, M, SN26

International students perceive Medical school not as culturally neutral, but as influenced by German cultural norms. In consequence many study participants mention cultural misunderstandings and frictions during interaction with their peers. 

When you don't drink alcohol [...] or prefer not to have physical contact with girls. Then [...] communication a lot harder. - Non-EU, M, SN9

Third world countries are different than it is here [Germany]. And here it is not like back there. There are so many differences in daily life. It takes time to change your thoughts, to accustomize to the life in Germany. - Non-EU, M, SN5

#### Dealing with integration

Many international students describe strategies to manage their own integration, which indicate a conscious handling of the contextual conditions mentioned above. Particularly active strategies are the search for meeting spaces to create new contacts and the creative use of their own resources in order to offer mutual benefits for contacts (see Figure 3 (Fig. 3)). 

Author: We talked before about investments. Do you need to do things for your network? 

SN14: Well it is about giving and taking. I cook them food and they bring me the knowledge - EU, F, SN14

Participants associated closeness between ones’ own culture and the German culture with an easier integration into the community, as well as previous experience with intercultural contacts. The latter indicates that intercultural experience may have a positive effect on integration in medical school.

Mhm, it depends a LOT on the nationality. It can happen that it plays no role. [...] We have one from [EU-Country 1] and one from [EU-Country 2] and I would say they are even more German than I am. - EU, F, SN18

I met really nice people and this is why it was so much fun at the beginning. But well of course I lived already two years in Germany. That is why I knew this already a bit - EU, F, SN25

## Discussion

The present study investigates the perception of the development and maintenance of personal networks by German and international medical students. In the process, influencing factors on their perceptions are distinguished. In regard to the first research question, we show that German students perceive their network development generally more positively than international students. Influencing factors on this are: the different associations with the groups of “medical students” and “foreigners”, an unfavorable evaluation of the academic use of contacts with international students, the majority position of German students, a strong cultural influence on the academic environment and different strategies of international students in dealing with integration. Our results are in line with previous studies conducted in other countries [29], [44], [45]. Our results reproduce findings from English speaking countries on the integration of foreign medical students at a German medical school [8], [28] and make clear that reports on the concerns of these students at the start of medical school are justified [43]. They also show that the medical student community is more heterogeneous than often assumed and mutual support and cohesion cannot be expected by itself [18].

### Influence factor: group affiliation

The theories of social identity and self-categorization provide an established theoretical framework for understanding the differently experienced group affiliations [25], [26]. They are based on conscious categorization processes of individuals, who associate themselves with groups and thus extend their own identity with the identity of the group. In this process, the salience of group as well as the evaluation of group members in the categorization plays an important role. Thus, high salience and a positive self-image of group members promote self-categorization into these groups [33]. With this background, the self-categorization of all study participants into the group of medical students is understandable, since studying medicine has a pronounced influence on their lives and distinguishes them from other social groups. The status as a medical student also represents a positive social role and is therefore a desirable social group. However, the feelings of affiliation with the “domestic” and “foreign” student group differ strongly. German students do not categorize themselves as “domestic”, as this attribute shared with the majority of others. International students instead experience this separation very strongly. Also it is perceived with a negative connotation due to possible isolation and poorer academic performance.

#### Influence factor: cost-benefit consideration

Although the focus on academic performance in medical schools [42] is perceived by all medical students, international students in particular experience this as a disadvantage in social life. The statements on the low academic value of contacts with international medical students in our research contradict previous studies of Lovell's research on student communities in medical schools [18]. Although he also refers to comparable behavior among medical students, he reports only little evidence on the abandonment of students with academic struggles. Instead, his study displays a cooperative community, in which mutual work and support exist despite competition. Our results, however, indicate that this competition weakens contacts rather than promoting them. The observed abandonment from underachieving students may be an alternative explanation for quantitative network studies [29], [30]. It explains the link between the network position of medical students in their respective class and academic achievement. Currently this phenomenon is often described through the equalization of academic performance among befriended medical students due to mutual support [18], [29]. Our results instead suggest a previous selection of high performing students and an exclusion of low performing students. This process may therefore increase differences in academic performance and lead to a pronounced academic stratification of medical student communities.

#### Influence factor: Framework conditions in medical school

Both, the theory of social identity and our findings illustrate that the emergence of group affiliation is sensitive to the context in which it takes place [27]. The composition of the medical student community can influence this context. Here the large linguistic and cultural heterogeneity of international medical students [41] contrasts with the linguistic and cultural homogeneous majority of German medical students [42]. Given these circumstances, it can be assumed that German or professional cultural norms and expectations are rarely explicit [49]. Implicit knowledge is thus relevant, which means that students from different cultural backgrounds cannot use their potential to the same extent. This reveals limitations of the often alleged cultural neutrality [50] of medicine and medical education.

#### Limitations

The present study is limited by its location at a single university in a federal state in the eastern part of Germany. Nevertheless, the informal exchange with research groups from other universities indicated similar impressions at their locations. The number of interviews was limited by our comparatively small research team, although we found saturation in the data. The composition of the sample is also a limitation, as we had particular difficulty in reaching out for students from non-EU countries. Last but not least, our qualitative approach allows detailed insights into the field of research, but it is limited in the quantification of these insights due to our qualitative sampling method. Further research may overcome these limitations through surveys at other locations as well as through a more standardized and quantitative focus.

## Conclusions

Our findings indicate challenges in the social interaction among medical students. Nonetheless, they also show opportunities for intervention. In particular, the potential of a shared perception of an identity as medical students is impressive. An example of this is the long standing international cooperation of medical students is the International Federation of Medical Student Associations (IFMSA) [https://ifmsa.org/]. Local interventions could support such successful collaboration by promoting a shared identity among medical students at their universities. The same applies to the critical questioning of the existing contextual framework conditions of the medical school as well as the student body [51]. Last but not least, faculties have the opportunity to incorporate the cultural knowledge of international medical students into medical training through specific offers and reforms of the framework conditions of their educational programs. In the case of the UMR, the project RONIAmed was initiated by students, which is intended to support contacts between international and German medical students during the study entry phase. It is currently being tested.

## Funding

The present study was supported by funds from the Prorector for academics and teaching (PSL-UMR-1-16) as well as a doctoral scholarship from the University of Rostock.

## Acknowledgements

We thank all participating students for their willingness to give us an insight into their experiences at UMR. In addition, we would like to thank the members of the student research group on Medical Education at the Institute of Immunology for their critical comments during data analysis.

## Competing interests

The authors declare that they have no competing interests. 

## Figures and Tables

**Table 1:**
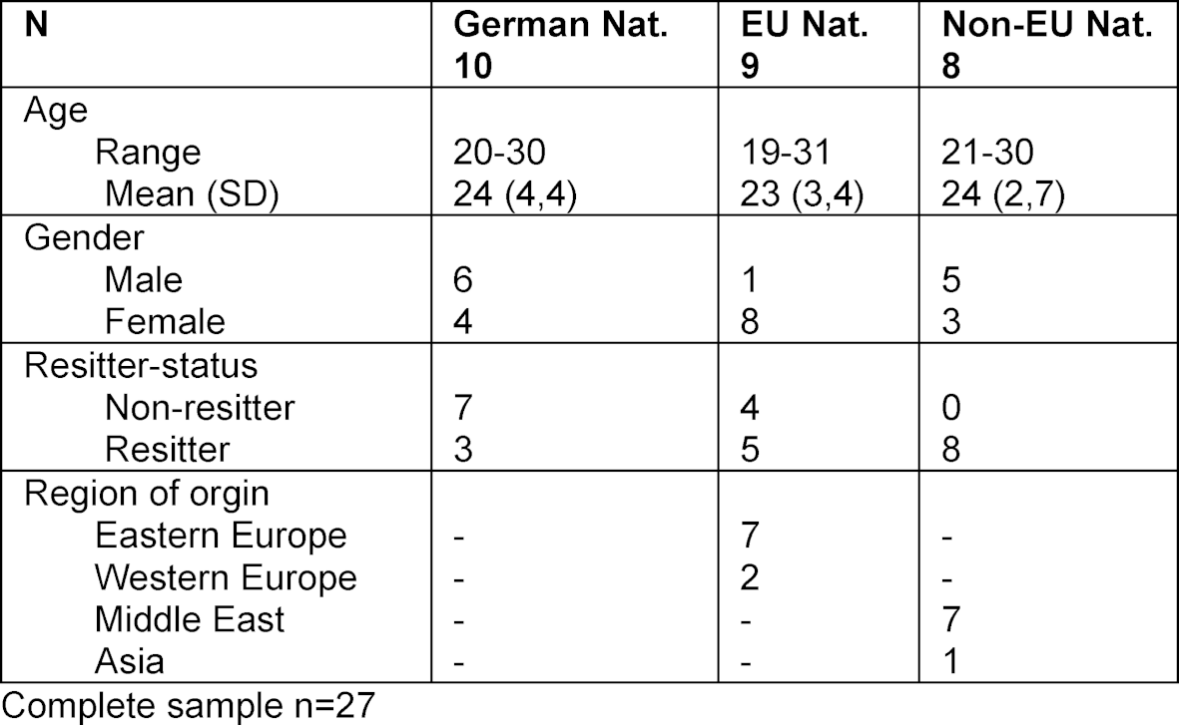
Sociodemographic information of the study participants based on nationality
